# Characterization of Flavor Profile of Sauced Pork from Different Regions of China Based on E-Nose, E-Tongue and Gas Chromatography–Ion Mobility Spectroscopy

**DOI:** 10.3390/molecules29071542

**Published:** 2024-03-29

**Authors:** Haibin Yuan, Huachang Wu, Mingfeng Qiao, Wanting Tang, Ping Dong, Jing Deng

**Affiliations:** 1 Cuisine Science Key Laboratory of Sichuan Province, Sichuan Tourism University, Chengdu 610100, China; 15883250273@163.com (H.Y.); mfqiao@163.com (M.Q.); twt15390399773@sina.com (W.T.); 2 Faculty of Food and Biological Engineering, Chengdu University, Chengdu 610106, China; 3 College of Food Science and Technology, Sichuan Tourism University, Chengdu 610100, China; whc3930590@sina.com

**Keywords:** sauced pork, volatile compounds, orthogonal partial least squares discriminant analysis, electronic nose, electronic tongue, classification

## Abstract

This study aimed to investigate the volatile flavor compounds and tastes of six kinds of sauced pork from the southwest and eastern coastal areas of China using gas chromatography–ion mobility spectroscopy (GC-IMS) combined with an electronic nose (E-nose) and electronic tongue (E-tongue). The results showed that the combined use of the E-nose and E-tongue could effectively identify different kinds of sauced pork. A total of 52 volatile flavor compounds were identified, with aldehydes being the main flavor compounds in sauced pork. The relative odor activity value (ROAV) showed that seven key volatile compounds, including 2-methylbutanal, 2-ethyl-3, 5-dimethylpyrazine, 3-octanone, ethyl 3-methylbutanoate, dimethyl disulfide, 2,3-butanedione, and heptane, contributed the most to the flavor of sauced pork (ROAV ≥1). Multivariate data analysis showed that 13 volatile compounds with the variable importance in projection (VIP) values > 1 could be used as flavor markers to distinguish six kinds of sauced pork. Pearson correlation analysis revealed a significant link between the E-nose sensor and alcohols, aldehydes, terpenes, esters, and hetero-cycle compounds. The results of the current study provide insights into the volatile flavor compounds and tastes of sauced pork. Additionally, intelligent sensory technologies can be a promising tool for discriminating different types of sauced pork.

## 1. Introduction

Sauced pork, a beloved Chinese cured and fermented meat product, is known for its distinctive flavor and taste. Traditionally made from pork belly or hind leg meat, it holds a special place among China’s national cured meats [1]. The production of traditional Chinese sauced pork involves four main steps: washing; salting; marinating with soy sauce, sweet noodle sauce, and spices; and then air-drying and maturation [2]. The flavor of sauced pork varies in different regions due to variations in marinades, curing time, and air-drying time. Marinades, consisting of sauces and spices, play a crucial role in meat processing. Each marinade provides a unique flavor, giving the meat products a distinct taste and texture. As the curing time increases, the flavor substances from the sauces and spices gradually penetrate the meat, influencing the flavor of the pork sauce. During the air-drying process, the microorganisms present in the raw materials release enzymes that oxidize and decompose organic substances like proteins, carbohydrates, and fats. These metabolic characteristics of bacteria cause changes in the flavor, as well as the physical and chemical properties, of the fermented meat products [3]. Sauced pork was primarily produced in Sichuan in the southwest, as well as in Shanghai, Zhejiang, and Anhui on the eastern coast of China. Each region has its own variation of marinade selection, marinating duration, air-drying time, and environmental factors, which may result in distinct flavors and further affect the choice of consumers for sauced pork. However, the research on sauced pork is mainly focused on the characterization of its physicochemical properties, volatile flavor components, and metabolic profiles under different high-pressure treatments during the process of marination in soy sauce [2,4]. The flavor characteristics of different kinds of sauced pork from distinct regions of China are still unknown.

Several studies have utilized different technologies such as gas chromatography–mass spectroscopy (GC-MS), E-noses, and E-tongues to investigate the flavor of meat products. These technologies offer highly sensitive and selective sensors to distinguish between food flavors. Shi et al. [5] employed solid-phase micro-extraction (SPME) coupled with GC-MS and an E-nose to assess the distinguishability of stewed pork ribs prepared in different ways. Du et al. [6] utilized SPME-GC-MS in conjunction with an E-nose and E-tongue to evaluate the flavor characteristics of bacon smoked with various wood chips. They observed significant effects of different smoking materials on the flavor and sensory attributes of the bacon. Furthermore, Zhang et al. [7] characterized the volatile compounds of golden pomfret fillets undergoing four drying methods using an E-nose, an E-tongue, and GC-MS. These techniques successfully distinguished the volatile compounds produced by different drying methods. In recent years, gas chromatography–ion mobility spectroscopy (GC-IMS) has emerged as a powerful detection technique with exceptional resolution and sensitivity for analyzing volatile flavor compounds of foods compared to GC-MS [8]. Food quality control, adulteration identification, flavor difference analysis, and the origin tracing of foods have been achieved according to the detection of flavor compounds by using GC-IMS [9,10,11,12,13].

In this study, we used the E-nose and E-tongue methods to detect and quantify the odors and tastes of sauced pork, respectively. The composition of the volatile flavor compounds was determined by GC-IMS and combined with relative odor activity value (ROAV) to determine the key flavor compounds of sauced pork. We also analyzed the sensory properties to further discriminate the characteristics of different kinds of sauced pork. In additional, a potential correlation between the volatile flavor compounds and the E-nose was discovered by using multivariate statistical analysis. The results of this study will provide a valuable reference for better understanding the mechanism of flavor formation of sauced pork and evaluating the feasibility of combining GC-IMS with an E-nose and E-tongue to identify different types of sauced pork from different regions of China.

## 2. Results and Discussion

### 2.1. E-Nose Analysis

**Figure 1:**
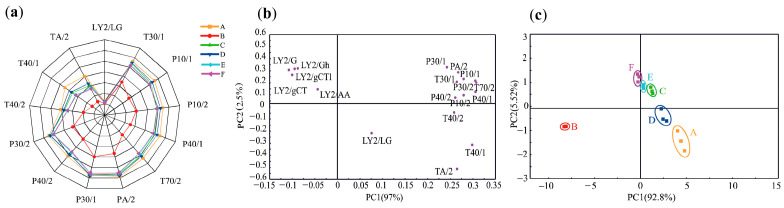
Radar chart of the electronic nose (E-nose) response data (**a**); principal component analysis loading plot (**b**) of the E-nose response data and score plot (**c**) of sauced pork.

The E-nose utilizes various sensitive sensors to detect and differentiate the aroma in a sample. It can detect subtle changes in volatile flavor compounds present in the sample [14]. As shown in Figure 1a, it was evident that certain sensors, such as T30/1, P10/1, P10/2, P40/1, T70/2, PA/2, P30/1, P40/2, P30/2, T40/2, T40/1, and TA/2, exhibit strong responses to the volatile compounds found in sauced pork. This suggests that sauced pork may contain a high abundance of aromatics, alkanes, and organic compounds. Significant differences (*p* < 0.05) were observed in the response values of different sauced pork varieties among the T30/1 (sensitive to polar compounds), T70/2 (sensitive to aromatic compounds), TA/2 (sensitive to organic compounds), P40/2 (sensitive to gases with strong oxidizing capacity), and T40/1 sensors. Compared to sample B, the response values of samples A, C, D, E, and F on each sensor were found to be stronger (*p* < 0.05), indicating that sample B had lower levels of alcohol and alkenes. Although all sauced pork samples exhibited a low response to the LY2/LG sensor, there were still variations in the response values among the different groups.

Principal component analysis (PCA) is not only useful for explaining sample differences, but also for extracting information about the variables that influence the spatial distribution of samples [15]. In Figure 1b,c, the PCA results of the E-nose are utilized to analyze the spatial distance and aroma distribution of sauced pork. PC1 and PC2 accounted for 92.8% and 5.52% of the total variance, respectively, capturing a significant portion of the odor information from the samples. The first principal component (PC1) shows a positive correlation with the LY2/LG, TA/2, T40/1, T40/2, and P10/2 sensors. Conversely, it demonstrates a negative correlation with the LY2/G, LY2/AA, LY2/Gh, LY2/gCT, and LY2/gCTI sensors.

The score plot in Figure 1c clearly demonstrates the separation of sauced pork samples from different regions. Sauced pork samples A and D from southwest China are located in the fourth quadrant, while samples B, C, E, and F from eastern coastal areas of China are distributed in the other quadrants. This indicates noticeable differences in the flavor characteristics of sauced pork from different regions of China. Among the different kinds of sauced pork from eastern coastal regions, samples C, E, and F were close to each other, indicating a similar odor between them, but were significantly different from sample B. Sensors TA/2 (ethanol), T40/1 (dimethyl disulfide), and LY2/LG (2-Methyl-3-furanthiol) appear to contribute more significantly to varieties A and D, suggesting that these two varieties contain higher levels of these substances. On the other hand, sensors P40/1 (furfuryl methyl disulfide), P40/2 (methyl mercaptan), P10/2 (n-heptane), and T30/1 (propanol) play a greater role in depicting the odor characterization of varieties C, E, and F. These findings highlight the efficacy of the E-nose as a valuable tool for discriminating between different regions of sauced pork. However, it should be noted that the specific differences between these samples may be challenging to comprehend solely using E-nose analysis.

### 2.2. E-Tongue Analysis

**Figure 2:**
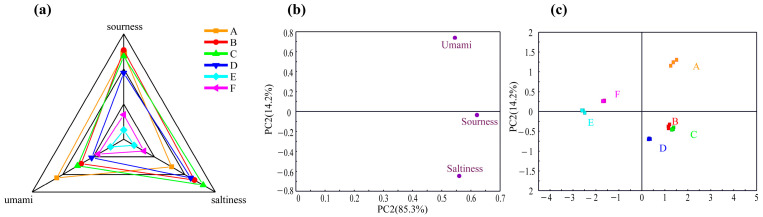
Radar chart of taste determination by the electronic tongue (**a**); principal component analysis loading plot (**b**) of the E-tongue response data and score plot (**c**) of sauced pork.

To further differentiate the flavor distinctions among various sauced pork varieties, the umami, salty, and sour tastes were analyzed using an E-tongue. The results displayed in Figure 2a indicate significant differences in umami, saltiness, and sourness among six different sauced pork varieties (*p* < 0.05), except for the sour taste between A and C (*p* > 0.05). These differences, especially in terms of sourness, may be attributed to the metabolic activity of lactic acid bacteria and the fermentation process of carbohydrates during the air-drying of sauced pork [16].

The PCA analysis (Figure 2b,c) revealed that PC1 and PC2 accounted for a cumulative contribution rate of 99.5%, suggesting that the E-tongue data sufficiently represented most of the taste information for different sauced pork varieties. In the load plot (Figure 2b), PC1 shows a positive correlation with sourness, saltiness, and umami, while PC2 exhibits a negative correlation with sourness and saltiness. As shown in Figure 2c, sample A is located in the first quadrant, indicating a significantly different taste from that of other samples. Samples B and C from eastern coastal areas exhibited similarity in taste to sample D from Sichuan, China, as they are all positioned within the fourth quadrant. Only E and F were positioned along the negative axis of PC1, but not in the same quadrant, indicating that they had their own unique flavor characteristics. In addition, samples B, C, and D exhibited relatively high levels of sour and salty tastes, while sample A possessed a relatively high umami taste. These findings imply that the E-tongue was not particularly effective in distinguishing sauced pork varieties from different regions. However, samples C, E, and F with the same odor are different in taste and can be distinguished by the E-tongue.

### 2.3. GC-IMS Analysis of Sauced Pork

As shown in Figure S1a, a total of 52 qualitative volatile compounds were detected from six different kinds of sauced pork. These compounds included eleven aldehydes (with a relative content ranging from 54.72% to 35.77%), eight esters (with a relative content ranging from 18.35% to 6.33%), seven terpenes (with a relative content ranging from 13.28% to 9.06%), six pyrazines (with a relative content ranging from 10.14% to 5.59%), six ketones (with a relative content ranging from 7.53% to 3.70%), three furans (with a relative content ranging from 3.71% to 0.76%), three alcohols (with a relative content ranging from 3.33% to 1.35%), one acid (with a relative content ranging from 0.39% to 0.13%), and seven other types of compounds (with a relative content ranging from 15.96% to 7.19%) (Table S1). It was obvious that the aldehydes were the most abundant compounds in sauced pork, both in terms of type and relative content. The formation of aldehydes in sauced pork mainly occurs via lipid degradation. The formed aliphatic aldehydes not only play an important role in contributing to the fatty flavor in sauced pork, but they also change the pathway of meat flavor compounds during the heating process [17,18]. Due to their low threshold, aldehydes are generally considered to be the main contributors to the flavor of meat products. Esters were also the main compounds in sauced pork, which could impart a fruity and creamy odor, mainly synthesized through β-oxidation and protein metabolism [19].

The addition of various spices and soy sauce during the marinade process significantly contributes to the production of terpenoids, alcohols, aldehydes, and furans in pork [2]. In this study, seven terpenes were detected in different types of sauced pork. Samples A and C exhibited the highest terpene content (13.06% and 13.29%, respectively), while sample F had the lowest content (9.06%). This difference may be attributed to the higher amounts of spices added during the curing process of samples A and C, as there is a strong correlation between terpenes and the flavor of spice-infused products [20]. Furans and pyrazines are also important flavor components in meat products. Pyrazines are usually generated via non-enzymatic chemical reactions and the condensation of two α-aminocarbonyl groups during the Strecker reaction, which could impart a roasted flavor [21]. Sample B had the highest relative content of pyrazines (10.14%), followed by sample D (9.83%), while the relative content of furans was relatively low among all kinds of sauced pork. The relative content of alcohol in sauced pork was the least significant. Alcohols are mainly derived through the reduction of aldehydes and the lipoxygenase pathway [22], such as 1-penten-3-ol (butter odor). Linear saturated alcohols have a high threshold and contribute minimally to the overall flavor of meat products [23,24].

To identify the difference of volatile organic compounds (VOCs) among the six kinds of sauced pork intuitively, a differential plot was generated using topographic plot deduction, with the A plot serving as the background. As illustrated in Figure S1b, the red vertical line represents the reaction ion peak (RIP), and each point on the right sides corresponds to a volatile organic compound [25]. A majority of blue spots were observed in sauced pork B, indicating that the content of most volatile flavor compounds in this variety was lower compared to other varieties. Conversely, there were more red spots in sample D, suggesting that the content of most volatile flavors was significantly higher than in other cultivars. Furthermore, there were also distinct substances present in cultivar D. The flavor of sauced porks B and D were significantly different from the others, and this result aligns with the findings from the E-nose analysis.

To further investigate the differences in volatile flavor compounds among different kinds of sauced pork, fingerprints were generated using a plug-in (Gallery Plot) of the instrument (Figure S1c). The VOC content varied considerably across the six varieties of sauced pork. Each column in Figure S1c represents the relative content of the same compound in different kinds of sauced pork. A darker red color indicates a higher concentration, while a lighter color indicates a lower concentration [26]. The fingerprint was divided into five regions based on the trend of content change. In region I, there were substances that were common to all sauced porks and present in high concentrations. These included butanal (mono and dimer), 2-methyl butanal-M, β-pinene, 2,3-butanedione, 2,6-dimethylpyrazine, and ethyl acetate. These compounds contribute fruity, woody, and barbecue flavors to pork sauce [27,28]. The volatile compounds in region II were the most abundant in sample A, including 2-methylbutanal-D, hexanal, and α-pinene (mono and dimer). This difference could explain the distinction between sample A and other kinds of sauced pork. Region III mainly consisted of substances with high content in sample B, such as 3-(methylthio) propanal, camphene, 1-penten-3-one, 2,5-dimethylfuran-M, cyclopentanone, and 2-butanone. Most of the substances in region IV were heterocyclic compounds that contribute to the unique meat flavor characteristics. The content of these compounds was highest in sample D compared to the other five varieties, and significant differences were observed (*p* < 0.05). Compounds in this region included 1-heptanol, (E)-2-hexenal, acetic acid, α-terpinene, 2-pentylfuran, 2-ethyl-5-methylpyrazine, 2-methylpyrazine, and 2-ethyl-3,5-dimethylpyrazine. The composition of flavor compounds in varieties C, E, and F was similar, primarily concentrated in region V. Notably, the contents of 1-penten-3-ol, n-heptanal, and myrcene in this region were higher and significantly different from other samples (*p* < 0.05). Overall, the comprehensive analysis of fingerprint spectra revealed differences in volatile flavor compounds among the different varieties of sauced pork. Samples A and B exhibited significantly lower types and contents of volatile compounds compared to the other four varieties, while sample D had higher levels. Samples C, E, and F showed similarities in the types and contents of substances.

**Figure 3:**
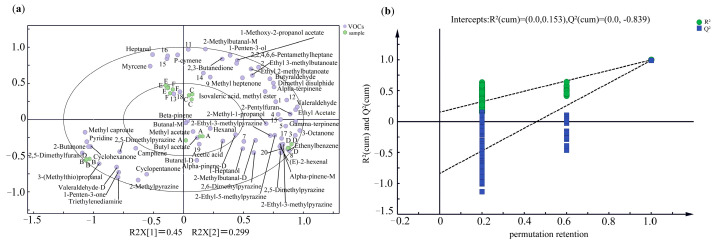
The biplot of OPLS–DA analysis (**a**) and permutation retention map (**b**) of volatile flavor compounds in different kinds of sauced pork.

To further investigate the aromatic properties of different types of sauced pork, an OPLS-DA model was conducted using 52 aromatic components as dependent variables and sauced pork types as independent variables. The model (Figure 3a) yielded acceptable fitting indices: RX^2^ = 0.981 for the independent variable, RY^2^ = 0.988 for the dependent variable, and Q^2^ = 0.975 for the prediction index. These results indicate that the OPLS-DA model demonstrates good reproducibility and predictability [29]. Validation of the model was performed through 200 permutation tests (Figure 3b). The intersection of the Q^2^ regression and the Y axis was negative, indicating that the model validation was successful. Therefore, the obtained results can be used to distinguish the characteristic substances of different types of sauced pork. Among the samples, varieties C, E, and F were concentrated along the midline of the first and second quadrants, suggesting that the flavors of these three varieties are similar. Samples A, B, and D were clearly separated along PC1. These results were similar to those of the E-nose. Samples A and D were distributed in the fourth quadrant, with the substances around D being significantly more abundant than the other samples. The main aroma components in these samples were butyl acetate, methyl acetate, 2,5-dimethylfuran, α-pinene, ethenyl benzene, (E)-2-hexenal, and 3-octanone. Sample B, on the other hand, was located in the third quadrant, with the main aroma components being 2,5-dimethylfuran, 3-(methylthio) propionaldehyde, 1-penten-3-ol, cyclohexanone, and 2-butanone. These results suggest that there are notable differences in the flavors of sauced pork between the southwest region and the eastern coastal region. Furthermore, certain differences of flavor substances were observed between different varieties of sauced pork in same region. Overall, these findings provide insight into the distinct aromatic properties of various types of sauced meat and highlight regional and varietal differences in flavor.

In the PLS-DA model, the variable importance in projection (VIP) value was calculated to determine the strength and impact of each flavor compound to the identification of different varieties of sauced pork. In general, substances with VIP > 1 were considered to contribute more to the discrimination of differences between samples [30]. In this study, a total of 13 compounds with a VIP value greater than 1 were identified, namely α-terpinene, β-pinene, propylene glycol monomethyl ether acetate, 1-pentene-3-one, isobutanol, 2-methyl butyraldehyde, 3-octane, butyraldehyde, dimethyl disulfide, styrene, methyl acetate, heptaldehyde, and n-valeraldehyde. These volatiles can be considered as flavor markers to distinguish between the six samples.

ROAV values could be used to screen key characteristic aroma compounds of different varieties of sauced pork [31]. According to Table 1, a total of seven compounds with ROAV values ≥ 1 were screened out of 52 volatiles, including 2-methylbutanal, 2-ethyl-3, 5-dimethylpyrazine, 3-octanone, ethyl 3-methylbutanoate, dimethyl disulfide, 2,3-butanedione, and heptane. Among these compounds, 2-methylbutanal, which can provide a strong roasted aroma, was proven to have the greatest impact on the flavor of sauced pork samples A, B, C, E, and F. However, 2-ethyl-3,5-dimethylpyrazine, with a baked potato flavor, had the greatest influence on the flavor of sample D compared with other samples [32,33]. The other substances provided butter, garlic, cheese, and fatty aromas for the flavor profile. Substances such as 1-methoxy-2-propanol acetate, 1-pentene-3-one, butanal-M, 2-methyl-1-propanol, and β-pinene have ROAV values ranging from 0.1 to 1. These substances play an important role in modifying the overall flavor of sauced pork [34]. In variety B, 1-pentene-3-one had an ROAV greater than one, while 3-octane had an ROAV less than one. However, the opposite was observed in the other five varieties. These two substances contribute buttery and aromatic herb flavors to sauced pork [32,35], indicating that they may be key substances responsible for the flavor difference between sample B and other varieties. In conclusion, the analysis of ROAV values provides valuable insights into the flavor characteristics of different sauced pork varieties and highlights the specific compounds responsible for the unique flavors observed.

### 2.4. Sensory Analysis

A total of 10 sensory properties classified into odor, appearance, taste, and mouthfeel were evaluated after a consensus among assessors. The results of the sensory evaluation are listed in Table 2. Among these samples, A, E, and F exhibited a stronger fatty aroma, clearer texture, greater elasticity, and higher redness and brightness, while sample B scored higher for meaty fragrance, sauce fragrance, and hardness. This discrepancy may be attributed to the difference in the fat-to-lean ratio of the raw materials. The high proportion of lean meat in sample B enhanced sauce adsorption during the marinating process, resulting in a more pronounced sauce fragrance and hardness, but with lower levels of elasticity and fatty aroma. Sample B had the lowest saltiness among all kinds of sauced pork, which is inconsistent with the result of the E-tongue. This disparity may be attributed to the saltiness masked by the higher level of sweetness. A meticulous assessment of the taste, aroma, and overall sensory properties reveals obvious differences in the total gustatory experience of various types of sauced meats.

### 2.5. Correlation Analysis between E-Nose and GC-IMS

**Figure 4:**
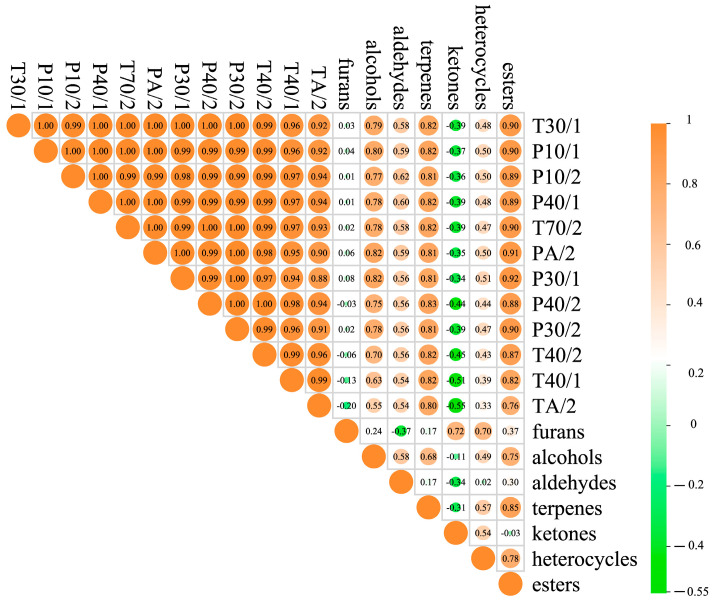
Correlations between electronic nose and volatile flavor components.

To further explore the relationship between volatile flavor compounds and odor, a Pearson correlation analysis was conducted between the GC-IMS data and the E-nose sensors (Figure 4). The E-nose sensors correlated well with alcohols, aldehydes, terpenes, and esters (r > 0.5, *p* < 0.05), indicating that the different flavor characteristics of sauced pork detected by the E-nose might be caused by the concentration differences in alcohols, aldehydes, terpenes, and esters [36]. Combined with the results of GC-IMS (Table S1), the E-nose sensors might be sensitive to α-pinene, heptanal, 1-Penten-3-ol, camphene, 2-methylbutana, γ-terpinene, 1-Heptanol, (E)-2-hexenal, myrcene, and heptanal, while showing negative correlations with ketones. These compounds cover almost all flavor substances of sauced pork, indicating that the E-nose can effectively describe the overall flavor of sauced pork and can be used to distinguish different kinds of sauced pork from different regions of China.

## 3. Materials and Methods

### 3.1. Samples

This study included samples of sauced pork sourced from six different regions, namely (A) Taibai sauced pork, (B) Jinhua sauced pork, (C) Wanlong sauced pork, (D) Sichuan flavor, (E) Huangshan sauced pork, and (F) Shanghai sauced pork. All samples were obtained through traditional handmade methods and purchased from Jindong Mall. Table 3 provides an overview of the ingredients listed on the product labels for each sauced pork sample examined in this study. Notably, sauced porks A and D originate from Sichuan, China, whereas sauced porks B, C, E, and F come from the eastern coastal region of China.

### 3.2. E-Nose Analysis

The E-nose analysis method used in this study was based on the work of Yu et al. [37] with slight modifications. The E-nose employed was the FOX 4000 (Alpha M.O.S., Toulouse, France). It consists of an array of 18 non-specific metal oxide sensors, which exhibit sensitivity to one or more specific substances. These sensors are evenly distributed within three matrix rooms of the mainframe. For the analysis, a 4 g sample of chopped sauced pork was accurately weighed and placed in a 10 mL headspace glass sampling vial. The vial was then heated using synthetic dry air at a temperature of 70 °C for a duration of 300 s. Subsequently, 1000 µL of the headspace was sampled with an injection flow rate of 1500 µL/s. The data acquisition parameters were set as follows: a data acquisition cycle of 1.0 s, an acquisition time of 120 s, an acquisition delay of 180 s, and an acquisition flow rate of 150 mL/min. To ensure reliability, parallel measurements were conducted 10 times, and the stable signal obtained by the sensor was analyzed, specifically focusing on the signal obtained 120 s after the last 3 measurements.

### 3.3. E-Tongue Analysis

In the E-tongue experiment, the Astree E-tongue (Alpha MOS, France) was utilized, equipped with a sixth set of sensors. The set included 7 specific sensors: AHS (sourness), PKS, CTS (saltiness), NMS (umami), CPS, ANS, and SCS. Ag/AgCl served as the reference electrode. The AHS (sourness), CTS (saltiness), and NMS (umami) sensors were selected to represent sour, salty, and umami taste, respectively. For the experiment, 50 g of sauced pork was chopped and mixed with 150 mL of de-ionized water. The mixture was homogenized in a homogenizer for 6 s, transferred to a 250 mL bottle, and filled with de-ionized water to a fixed volume. Next, ultrasonic extraction was conducted for 30 min. The extract was then rapidly filtered using filter paper to remove primary impurities. The resulting filtrate, 80 mL in volume, was transferred to a specialized beaker for E-tongue analysis. The E-tongue was operated under specific measurement conditions: a data acquisition time of 120 s, an acquisition cycle of 1.0 s, no acquisition delay, and a stirring speed of 1 r/s. Each sample was analyzed 5 times, and the stable values from the last 3 measurements were considered as the test results.

### 3.4. GC–IMS Analysis of Volatile Compositions

The GC–IMS analytical method used in this study was based on the method described by Guo et al. [38]. However, some modifications were made to the procedure. In this study, the volatile components in sauced pork were analyzed using a gas chromatography ion migration spectroscopy system called Flavor Spec^®^ (Gesellschaft für Analytische Sensorsysteme mbH, Dortmund, Germany). The system was equipped with an autosampler unit that allowed direct sampling from the headspace using an airtight heated syringe. The gas chromatography (GC) analysis was carried out using a capillary column with dimensions of 30 m × 0.53 mm × 1 μm (WAX column). To start the analysis, 1.5 g of sauced pork samples were placed in a 20 mL headspace glass sampling vial and incubated at 60 °C for 15 min. After incubation, 500 µL of headspace gas was sampled and automatically injected into the injector at a temperature of 85 °C under the splitless injection mode. The samples were then driven into the capillary column using nitrogen gas (purity ≥ 99.999%) at a programmed flow rate: 0 to 20 min at 2 mL/min, 2 to 10 min at 10 mL/min, and 10 to 40 min at 100 mL/min. The flow was then stopped. The resulting ions were directed into a 98 mm long drift tube, operating at a constant temperature of 45 °C and a voltage of 5 kV. The drift tube was filled with a drift gas (nitrogen, purity 99.99%) flowing at a rate of 150 mL/min. All analyses were conducted in triplicate to ensure the accuracy and reliability of the results.

### 3.5. Relative Odor Activity Value (ROAV)

In this study, the concentration of VOCs obtained by GC-IMS was expressed by the relative content (%); therefore, the ROAV (relative odor activity value) was measured to evaluate the specific contribution of each compound to the overall aroma. This method follows the principle described by the published literature [35,39]. By calculating the ROAV of each flavor compound, it is possible to determine the key flavor compounds present in the sample under investigation. Odor thresholds in the air of volatile flavor compounds were provided in [32].

ROAV = C_A_/T_A_ × T_stan_/C_stan_ × 100

During the test, C_A_ represents the relative percentage content of a particular compound, while T_A_ denotes the threshold concentration of that compound measured in mg/kg. Furthermore, C_stan_ represents the relative content of the substance contributing the most to flavor, expressed as a percentage, while T_stan_ represents the threshold concentration (in mg/kg) of the substance that has the greatest impact on flavor.

### 3.6. Sensory Evaluation

The sensory evaluation in this study was based on a method previously described [40,41], with slight modifications. Ten volunteers, containing five men and five women from Chengdu University who had received sensory training in food evaluation, participated in the evaluation of the organoleptic properties of the sauced pork. In order to familiarize the researchers with the sauced pork samples, we conducted three training sessions and one test. In the initial two training sessions, the assessors scrutinized the descriptive terminology pertaining to the appearance, flavor, and taste of the sauced pork. Subsequently, during the third session, a consensus was reached by reconfirming previously disputed descriptions. The evaluation was conducted in individual sensory evaluation booths, where participants were asked to score sample based on aroma, visual appearance, and taste by using a 100 mm line scale (0–25 being poor; 25–50 being moderate; 50–75 being good; 75–100 being excellent). Furthermore, participants were asked to make a choice about their overall satisfaction with the sauced pork (scored from 0 to 5).

### 3.7. Statistical Analysis

The data of GC-IMS were analyzed qualitatively and semi-quantitatively using the VOCal software 5.3 of the Flavour Spec^®^ flavor meter. The Reporter and Gallery Plot plug-ins were used to compare the spectral differences between samples and to form a fingerprint, respectively.

For data analysis and visualization, IBM SPSS Statistics 26 and Origin 2017C 64-bit software were utilized. The Duncan method, along with significance difference analysis and analysis of variance tests, was employed to determine differences between varieties. Correlation analysis and data visualization were performed using the website https://www.chiplot.online/ (accessed on 24 September 2023). Principal component analysis and orthogonal partial least squares discriminant analysis were conducted using the SIMCA14.1 software.

## 4. Conclusions

In this study, the flavor characteristics of six kinds of sauced pork from the eastern coastal area and southwest of China were investigated. The results showed that the odor and taste of different kinds of sauced pork had certain differences, which could be effectively distinguished by an E-nose combined with an E-tongue. Through GC-IMS analysis, a total of 52 volatile compounds were identified, including eleven aldehydes, eight esters, seven terpenes, six pyrazines, six ketones, three furans, three alcohols, one acid, and seven other types. A total of 13 key compounds (ROAV ≥ 0.1) were obtained from 52 volatiles. The ROAV showed that sauced pork from the southwest region exhibited a stronger butter aroma, while the fat aroma was slightly weaker when comparing sauced pork from the southwest region to that of the eastern coastal areas. OPLS-DA multivariate data analysis discovered 13 volatile compounds (VIP > 1), such as α-terpinene, β-pinene, propylene glycol monomethyl ether acetate, and 1-pentene-3-one, which served as the flavor markers to distinguish the six samples. The results of the sensory evaluation showed that varieties B, C, E, and F had higher acceptance. Furthermore, a strong correlation was observed between the E-nose sensor and volatile flavor compounds. In conclusion, this study provides a theoretical basis for distinguishing the origin of sauced pork and the flavor formation mechanism. The findings highlight the potential of using a combination of analytical techniques to explore and differentiate flavor profiles in food products.

## Figures and Tables

**Table 1:** Key volatiles (ROAV ≧ 0.1) in six groups of sauced pork.

Compound	Threshold (mg/kg)	ROAV						
		A	B	C	D	E	F	Odor Description
2-Ethyl-3,5-dimethylpyrazine	0.00004	28.91	31.94	24.46	100.00	61.76	37.06	Baked potatoes, burnt flavor
β-pinene	0.180	0.19	0.16	0.12	0.12	0.06	0.10	Pine wood fragrance
1-Methoxy-2-propanol acetate	0.016	0.64	<0.10	0.89	0.66	0.31	0.68	-
1-Penten-3-ol	0.001	0.26	2.66	0.14	0.70	0.33	0.16	Butter fragrance
2-Methyl-1-propanol	0.033	0.33	0.14	0.13	0.35	0.16	0.13	-
2-Methylbutanal-M	0.001	100.00	100.00	100.00	100.00	46.67	100.00	Apple aroma, strong roasting smell
3-Octanone	0.0013	6.73	0.87	4.75	16.99	7.93	4.77	Butter and grass fragrance
Butanal-M	0.100	0.61	0.96	0.45	0.81	0.38	0.72	Fruit aroma
Dimethyl disulfide	0.008	2.97	1.50	3.67	4.34	2.13	3.10	Garlic, onion
Ethenyl benzene	0.15	0.10	<0.10	<0.10	0.41	<0.10	<0.10	Sweet, floral
2,3-Butanedione	0.00018	26.73	18.24	22.76	11.44	27.15	23.98	Butter and cheese
Heptanal	0.001	22.15	36.70	45.49	7.96	73.63	87.15	Apple, fat fragrance
ethyl 3-methylbutanoate	0.000069	4.94	2.14	8.84	6.45	12.84	7.42	Fruit

**Table 2:** Sensory evaluation results of sauced pork.

Attributes		A	B	C	D	E	F
Odor	Fatty	73.7 ± 2.86 ^a^	55.4 ± 2.58 ^c^	61 ± 3.06 ^b^	58.1 ± 3.07 ^bc^	64.8 ± 2.32 ^b^	65.1 ± 2.53 ^b^
	Meaty fragrance	66.1 ± 3.14 ^c^	79.2 ± 3.08 ^a^	72.2 ± 2.10 ^b^	73.5 ± 2.74 ^b^	72.7 ± 3.31 b^b^	71.7 ± 4.11 ^b^
	Sauce fragrance	61.5 ± 2.30 ^d^	82.1 ± 3.84 ^a^	74.1 ± 2.32 ^b^	74.5 ± 2.22 ^b^	66.2 ± 2.84 ^c^	68.4 ± 3.17 ^c^
Appearance	Texture clarity	81.4 ± 3.13 ^ab^	57.2 ± 3.39 ^d^	70.1 ± 2.78 ^c^	55.9 ± 4.31 ^d^	78.5 ± 3.50 ^b^	85.4 ± 4.20 ^a^
	Redness and brightness	75.5 ± 3.69 ^b^	48.0 ± 3.43 ^d^	56.7 ± 2.98 ^c^	49.1 ± 5.30 ^d^	78.1 ± 3.70 ^b^	84.8 ± 3.94 ^a^
Taste	Sweet	48.6 ± 3.47 ^e^	87.2 ± 4.21 ^a^	54.5 ± 3.69 ^d^	63.6 ± 5.27 ^c^	47.4 ± 5.40 ^e^	69.6 ± 3.86 ^b^
	Salty	75.3 ± 4.06	51.3 ± 5.81	79.1 ± 3.76	86.3 ± 3.95	53.8 ± 5.01	53.5 ± 5.44
Mouthfeel	Hardness	47.2 ± 2.35 ^e^	75.7 ± 3.71 ^a^	59.2 ± 2.66 ^c^	66.2 ± 2.44 ^b^	45.4 ± 3.41 ^e^	53 ± 4.64 ^d^
	Elasticity	59.7 ± 5.66 ^b^	45.4 ± 3.47 ^c^	47.7 ± 4.4 ^c^	47.8 ± 3.22 ^c^	57.5 ± 5.06 ^b^	73.4 ± 5.64 ^a^
	Juicy	56.9 ± 4.63	40.9 ± 5.74	50.3 ± 3.5	46.5 ± 3.24	56.3 ± 3.74	67.4 ± 4.77
Overall satisfaction		2.8 ± 0.33	4.6 ± 0.42	3.1 ± 0.34	3.1 ± 0.27	2.8 ± 0.25	3.1 ± 0.29

Note: Different superscript letters in the same row indicate significant differences (*p* < 0.05).

**Table 3:** The ingredient information of different kinds of sauced pork.

Group	Ingredient List	Preservation Conditions
A	Pork belly with sweet noodle sauce, salt, sugar, monosodium glutamate, spices, liquor, sodium nitrite, and D-sodium erythorbate.	0~4 °C 180 d
B	Pork belly with salt, brewed soy sauce, sugar, monosodium glutamate, edible essence, monascorubin, sodium nitrite, and D-sodium erythorbate.	0~4 °C 180 d
C	Pork belly with salt, brewed soy sauce, sugar, monosodium glutamate, liquor, and sodium nitrite.	0~4 °C 30 d
D	Pork belly with salt, spices, sweet noodle sauce, sugar, sodium glutamate, and sodium nitrite.	0~4 °C 180 d
E	Pork belly with brewed soy sauce, sugar, salt, liquor, monosodium glutamate, spices, D-sodium erythorbate, and sodium nitrite.	−10 °C 365 d
F	Pork belly with sugar, salt, brewed soy sauce, liquor, monosodium glutamate, sodium nitrite, monascorubin, and ethyl maltol.	0~4 °C 30 d

## Data Availability

All data can be provided as needed.

## Supplementary Materials

The following supporting information can be downloaded at https://www.mdpi.com/article/10.3390/molecules29071542/s1.
