# Supplementary material for: Characterization of Flavor Profile of Sauced Pork from Different Regions of China Based on E-Nose, E-Tongue and Gas Chromatography–Ion Mobility Spectroscopy
Source: Molecules. 2024 Mar 29;29(7):1542. doi: 10.3390/molecules29071542 (PMC11013253; doi:10.3390/molecules29071542)
Supplement: Supplementary file 1 [file molecules-29-01542-s001.zip › molecules-2859519-supplementary.pdf]

Table S1. Volatile flavor compounds identified from different kinds of sauce pork

Category	Chemical compound	Odor description	CAS	Molecular formula	RI	RT	Peak volume					
							A	B	C	D	E	F
Pyrazines	2,6-Dimethylpyrazine	Roast beef, nut aroma	108-50-9	$C_6H_8N_2$	1347.8	1176.692	4094.520±917.596	3600.966±437.164	3242.848±199.143	4977.403±528.528	3760.574±324.081	3498.336±87.575
	2,5-Dimethylpyrazine	Roast beef, nut aroma	123-32-0	$C_6H_8N_2$	1347.6	1175.981	1600.107±122.562	1456.454±153.659	1114.526±63.099	893.293±94.464	1084.206±65.673	1037.345±45.345
	2-Ethyl-3-methylpyrazine	Fruity aroma	15707-23-0	$C_7H_{10}N_2$	1435.1	1438.241	284.28±41.059	117.075±5.998	218.538±47.134	1676.968±80.98	380.487±231.992	183.1±16.031
	2-Ethyl-5-methylpyrazine	Fruity aroma	13360-64-0	$C_7H_{10}N_2$	1427.6	1415.843	84.709±16.004	61.942±7.173	91.942±39.646	392.476±124.47	159.234±63.559	105.359±5.335
	2-Methylpyrazine	Aromas of nuts and cocoa	109-08-0	$C_5H_6N_2$	1267.1	925.073	103.478±22.633	284.373±34.914	87.635±14.091	160.788±9.507	71.78±9.07	63.173±1.358
	2-Ethyl-3,5-dimethylpyrazine	-	13925-07-0	$C_8H_{12}N_2$	1479.8	1572.308	167.335±31.333	142.951±13.039	179.287±40.253	1191.15±432.831	452.568±264.561	265.577±48.714
Alcohols	2-Methyl-1-propanol	-	78-83-1	$C_4H_{10}O$	1098	462.240	1570.612±82.549	515.437±52.255	792.813±63.933	1588.746±30.051	1495.543±104.882	739.718±16.195
	1-Penten-3-ol	Butter fragrance	616-25-1	$C_5H_{10}O$	1144.8	567.771	955.375±85.002	183.507±10.399	1645.087±86.352	1364.939±187.434	2184.668±131.754	1866.675±59.338
	1-Heptanol	-	111-70-6	$C_7H_{16}O$	1427.5	1415.573	28.95±2.581	53.848±8.18	25.828±10.132	130.383±70.247	57.824±26.876	28.115±3.482
Furans	2,5-Dimethylfuran-M	-	625-86-5	C_6H_8O	925.8	303.577	420.277±66.04	1914.371±89.917	273.508±16.135	97.955±8.088	609.564±47.099	397.384±4.845
	2-Pentylfuran	Fragrance of flowers and fruits	3777-69-3	$C_9H_{14}O$	933.1	307.760	234.195±55.837	99.525±8.912	331.567±2.781	707.844±57.262	355.202±72.135	371.19±12.06
	2,5-Dimethylfuran-D	-	625-86-5	C_6H_8O	1237.6	826.845	118.444±66.098	56.381±5.931	39.965±1.02	571.113±13.528	59.17±5.61	46.237±0.532
Aldehydes	3-(Methylthio)propanal	-	3268-49-3	C_4H_8OS	1436.7	1443.002	379.13±106.482	666.067±69.59	310.673±73.814	172.528±7.366	176.672±6.649	209.23±18.519
	Heptanal	Fat aroma	111-71-7	$C_7H_{14}O$	1187.8	665.006	3205.557±214.069	4105.981±101.011	8335.878±792.734	2369.013±187.76	13488.73±1345.021	15612.527±745.51
	Butanal-M	Fruity aroma	123-72-8	C_4H_8O	826.3	246.518	8816.764±987.009	10781.924±466.25	8236.837±663.632	11312.455±86.919	9358.785±73.461	12893.877±136.415
	Butanal-D	Fruity aroma	123-72-8	C_4H_8O	808.9	236.513	73.767±27.603	78.208±13.568	82.544±21.818	74.13±13.555	37.549±4.84	28.999±0.832
	Valeraldehyde-M	Almonds, spicy	110-62-3	$C_5H_{10}O$	1000.9	349.498	893.695±69.28	1037.657±4.885	516.85±64.786	300.24±3.997	189.082±20.808	392.518±4.829
	Valeraldehyde-D	Almonds, spicy	110-62-3	$C_5H_{10}O$	1002.1	350.797	2303.241±389.213	375.068±43.052	2501.317±98.847	3765.476±63.065	2534.329±74.649	1699.817±89.909
	Hexanal	Apple, fat fragrance	66-25-1	$C_6H_{12}O$	1095.8	458.483	572.854±17.162	223.402±13.805	184.043±13.92	345.76±37.877	501.438±197.86	191.234±15.492
	2-Methylbutanal-M	-	96-17-3	$C_5H_{10}O$	883.1	279.047	14469.191±144.832	11188.838±290.764	18323.075±166.651	13898.955±54.646	18320.548±149.742	17913.882±97.368
	2-Methylbutanal-D	-	96-17-3	$C_5H_{10}O$	887	281.317	473.742±20.645	80.698±3.802	137.005±7.768	331.53±19.107	119.492±7.937	221.141±7.128
	(E)-2-hexenal	Fragrant grass	6728-26-3	$C_6H_{10}O$	1221.8	774.097	16.881±2.327	16.72±1.017	23.987±4.421	149.004±13.067	21.62±3.442	19.064±2.133
	Butyraldehyde	Fragrant grass and fruit	123-72-8	C_4H_8O	883.1	279.078	986.723±30.478	262.945±7.128	1122.449±26.173	1099.197±59.705	827.402±42.151	1216.736±9.342
Acid	Acetic acid	Spicy, sour	64-19-7	$C_2H_4O_2$	1477.4	1565.253	170.981±30.634	219.837±14.657	112.217±30.853	339.888±96.549	272.411±129.193	186.711±27.748
Terpenoids	α-terpinene	Lemon scent	99-86-5	$C_{10}H_{16}$	1237.9	827.596	3349.504±213.501	998.954±202.255	5331.359±68.416	4481.47±261.757	3396.995±360.697	3229.151±283.598
	β-pinene	Pine wood incense	127-91-3	$C_{10}H_{16}$	1282.8	977.468	4818.758±294.784	3210.644±12.982	3894.022±50.025	3061.843±192.97	3492.109±43.356	3145.65±102.534
	α-pinene-M	Pine wood incense	80-56-8	$C_{10}H_{16}$	1036	389.796	329.798±63.244	30.725±2.671	51.611±3.226	161.799±1.538	134.529±33.341	81.691±3.647
	α-pinene-D	Pine wood incense	80-56-8	$C_{10}H_{16}$	941.8	312.719	226.715±13.628	69.521±5.555	79.702±7.612	272.409±17.79	132.324±19.247	115.373±1.897
	γ-terpinene	Bitter, citrus taste	99-85-4	$C_{10}H_{16}$	853.6	262.159	401.067±59.441	206.853±27.897	598.622±10.342	921.947±35.919	410.517±10.949	422.26±33.607
	Myrcene	A light aroma of balsam	123-35-3	$C_{10}H_{16}$	1335.6	1139.954	376.892±23.674	343.752±6.575	353.161±6.978	238.385±27.578	988.031±50.341	724.197±6.302
	Camphene	Camphor flavor	79-92-5	$C_{10}H_{16}$	1190.2	670.506	506.998±29.282	1701.202±31.503	985.224±54.929	1142.784±54.51	1215.473±90.758	628.791±20.175
Ketones	3-Octanone	Butter fragrance	106-68-3	$C_8H_{16}O$	1112.5	494.951	1265.051±81.089	126.338±22.949	1131.179±81.792	3069.33±52.567	857.613±112.445	1110.741±54.497
	Cyclohexanone	Earthy smell	108-94-1	$C_6H_{10}O$	1023.2	375.051	785.748±286.834	1050.762±175.064	526.597±53.224	381.642±40.439	698.299±65.381	555.371±44.437
	1-Penten-3-one	Mushroom and metal smell	1629-58-9	C_5H_8O	1022.6	374.349	37.232±5.12	297.583±15.009	24.741±1.92	97.589±3.421	41.915±5.625	29.037±2.1
	2,3-Butanedione	Earthy smel	431-03-8	$C_4H_6O_2$	1144.7	567.568	696.218±156.744	367.377±22.743	750.8±23.669	613.42±18.618	895.231±22.856	773.106±8.497
	2-Butanone	Aroma, fruity	78-93-3	C_4H_8O	1046.6	401.986	617.408±28.302	2346.22±124.277	632.315±7.213	662.722±27.397	878.554±39.689	1298.701±38.137
	Methyl heptenone	Citrus flavor	110-93-0	$C_8H_{14}O$	1266.4	922.954	46.947±4.258	19.188±3.232	81.506±9.614	116.699±8.238	156.88±25.125	84.528±5.655
Heterocyclics	Ethenyl benzene		100-42-5	C_8H_8	1200.8	703.771	2063.495±163.969	159.705±5.554	847.219±97.259	8462.388±126.195	541.907±144.605	912.598±78.449
	Pyridine	Unpleasant smell	110-86-1	C_5H_5N	1236.6	823.377	275.222±15.695	1069.651±129.731	542.818±25.93	302.895±25.484	418.793±19.142	509.557±12.861
	Triethylenediamine	-	280-57-9	$C_6H_{12}N_2$	1505.3	1648.948	466.929±15.854	686.757±40.301	328.963±53.68	319.552±13.294	247.709±16.289	225.975±8.479
	Dimethyl disulphide	Unpleasant smell	624-92-0	$C_2H_6S_2$	1040.4	394.868	3614.541±598.547	1405.932±87.058	5645.986±46.027	5072.503±146.05	3448.98±96.468	4657.98±3.437
	Cyclopentanone	Mint flavor	120-92-3	C_5H_8O	943.8	313.913	123.152±45.362	367.916±39.73	165.416±26.198	242.301±51.649	117.632±23.486	111.072±5.578
	2,2,4,6,6-Pentamethylheptane	-	13475-82-6	$C_{12}H_{26}$	1200.8	703.822	220.897±62.476	53.932±0.378	357.349±22.712	207.978±2.112	248.227±15.226	274.087±6.115
	p-Cymene	Citrus flavor	99-87-6	$C_{10}H_{14}$	1244.8	850.878	344.887±42.051	268.903±10.31	763.716±57.445	478.66±7.72	1504.553±270.778	1348.158±24.834
Esters	Ethyl Acetate	Pineapple aroma	141-78-6	$C_4H_8O_2$	845.7	257.612	11187.618±801.908	2442.91±123.519	9350.367±58.799	13071.371±115.954	7409.896±165.926	9287.858±111.322
	Methyl acetate	Fragrant grass	79-20-9	$C_3H_6O_2$	807.9	235.924	214.952±51.972	237.44±17.737	298.571±33.917	231.312±13.773	214.899±11.958	146.656±0.628
	1-Methoxy-2-propanol acetate	-	108-65-6	$C_6H_{12}O_3$	1236.3	822.490	1474.029±11.71	155.319±89.176	2621.918±92.291	1477.427±34.825	1967.101±223.479	1961.322±75.24
	ethyl 3-methylbutanoate	Fruity aroma	108-64-5	$C_7H_{14}O_2$	1063.4	421.235	49.316±7.236	16.492±3.581	111.734±4.992	132.522±6.587	162.31±13.192	91.717±4.974
	Methyl caproate	Ester aroma, fruity aroma	106-70-7	$C_7H_{14}O_2$	1200.6	703.148	195.758±6.225	420.194±6.95	323.144±22.265	150.807±17.681	217.93±19.343	240.07±26.916
	isovaleric acid, methyl ester	Fruity aroma	556-24-1	$C_6H_{12}O_2$	1014.9	365.544	122.663±26.371	51.698±3.141	136.385±6.688	294.057±18.767	286.433±25.423	197.028±10.661
	ethyl 2-methylbutanoate	Fruity aroma	7452-79-1	$C_7H_{14}O_2$	1046.6	401.929	495.472±53.179	161.064±26.33	1106.563±18.108	901.176±11.811	1087.883±29.842	704.977±25.482
	butyl acetate	Fruity aroma	123-86-4	$C_6H_{12}O_2$	1095.4	458.097	328.697±34.003	51.929±5.623	43.202±4.416	66.483±11.746	91.429±7.649	50.146±5.596

Note: All values are the mean ± standard deviation of three replicates. RI represents retention index and RT represents retention time.

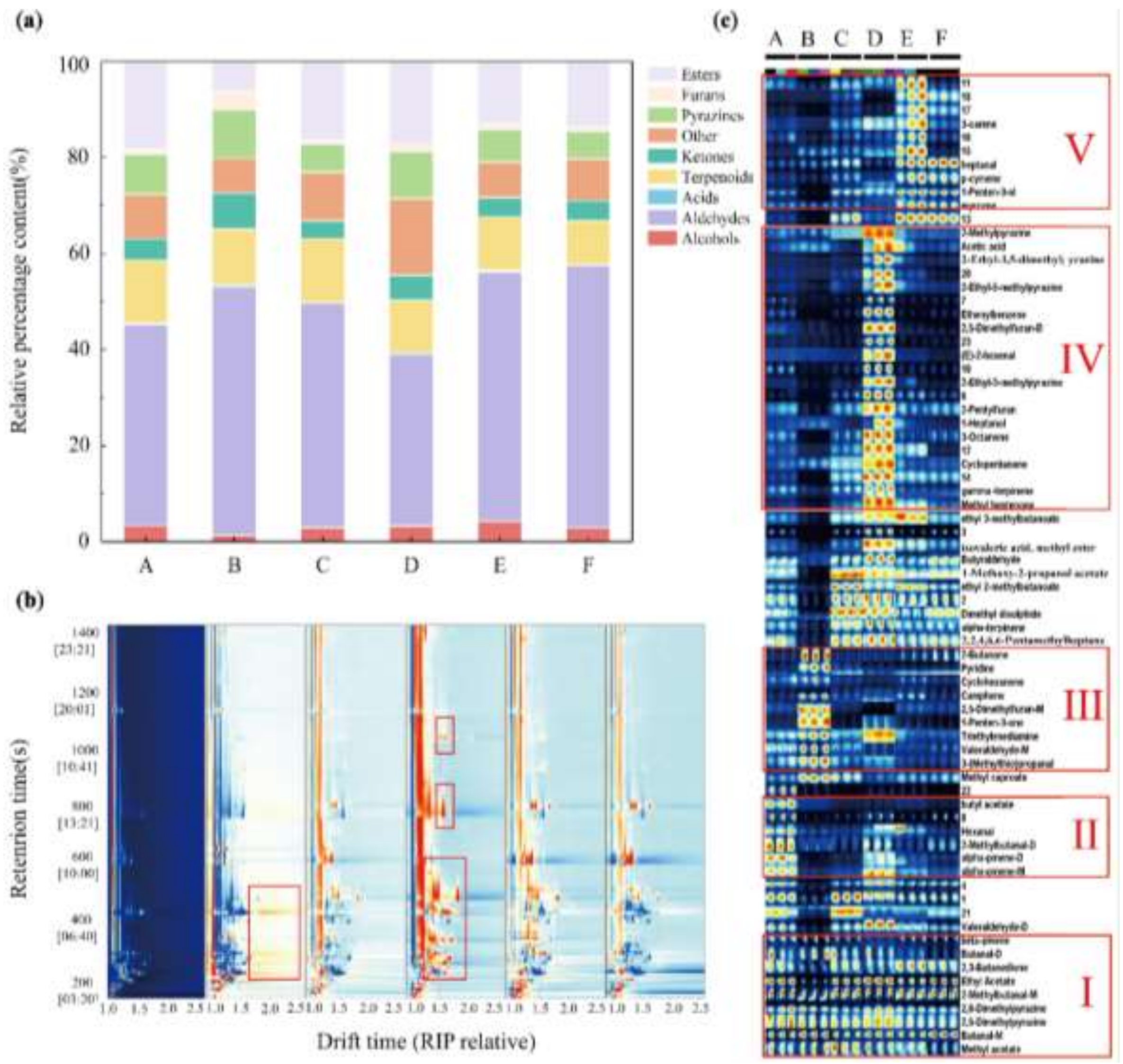

Figure S 1. GC–IMS observations of different kinds of sauced pork. (a) Chart of the relative percentage content of different kinds sauced pork based on the peak volume of VOCs. (b) Comparison of GC-IMS spectra of VOCs in sauced pork from six different varieties (after color deduction). (c) Fingerprint spectra for volatile compounds identified in sauced pork by GC–IMS.
